# New Insights in Canine Reproduction

**DOI:** 10.3390/ani11072021

**Published:** 2021-07-06

**Authors:** Monica De los Reyes, Nucharin Songsasen

**Affiliations:** 1 Laboratory of Animal Reproduction, Department of Animal Production, Faculty of Veterinary Sciences, University of Chile, Santiago 8820000, Chile; 2 Center for Species Survival, Smithsonian Conservation Biology Institute, 1500 Remount Rd., Front Royal, VA 22630, USA; songsasenn@si.edu

Reproduction in canids has been characterized by having some peculiar aspects, such as the extended reproductive cycle and ovulation of immature oocytes. However, progress toward the understanding of mechanisms underlying these unique features has been very slow, which, in turn, limits our ability to control or enhance reproduction and to develop clinical tools to diagnose reproductive disorders. The Special Issue assembles research and review articles focusing on various aspects of canine reproduction, including reproductive diversity, semiochemical communication, mechanisms regulating oocyte maturation, germ cell development, reproductive disorders, pregnancy, and influence of anesthetic drugs on ovarian blood flow.

(i). Reproductive diversity: The canine family comprises a wide variety of species, some of which are listed as endangered by the International Union for Conservation of Nature. Reproduction is fundamental in animal conservation programs, and in this regard, the knowledge of different reproductive characteristics is fundamental to apply the various strategies to ensure the genetic and demographic sustainability of a population [1]. A review paper presented in this Special Issue summarizes the current understanding of canid reproduction, including estrus cyclicity, seasonality, and seminal traits, with the emphasis on species diversity and also the application of reproductive technologies in wild canid conservation. 

(ii). Semiochemical communication: Similar to other species, male canids distinguish the reproductive status of conspecific females based on odor [2]. A study on semiochemical communication between female and male dogs demonstrated that estrous urine contained non-volatile compounds that required direct contact (smell and lick) to provoke sexual interest/behaviors in male dogs. 

(iii). In vitro oocyte maturation: One of the most intriguing characteristics of canine reproduction is the ovulation of immature oocytes that require an additional 48–72 h within the oviduct to acquire fertilizing and developmental abilities [3]. To date, the efficiency of in vitro maturation (IVM) protocols in canids is very low (<20% on average). The Special Issue features one review and three research articles focusing on mechanisms regulating dog oocyte maturation and development. Specifically, a review paper summarizes the crucial role of exosomes in regulating reproductive processes and discusses potential mechanisms in regulating dog oocyte maturation and fertilization. A study on in vitro production of extracellular vesicles from cultured dog oviductal cells has identified close to 400 proteins involved in the canonical pathways with essential functions in oocyte and embryo development. Such findings could serve as an important foundation for the development of improved in vitro culture conditions for IVM and in vitro fertilization. Further, a study on the expression of different genes associated with oocyte-embryo development at a different stage of the reproductive cycle has demonstrated that progesterone receptor, cyclooxygenase-2, growth differentiation factor-9, and bone morphogenetic protein-15 are differentially expressed in a stage-dependent manner in oviduct, indicating the importance of hormonal regulation in the oviductal function that, in turn, influences gamete maturation, fertilization, and embryonic development. Finally, a study has shown that supplementation of L-carnitine during IVM improves nuclear maturation and fertilization rates of dog oocytes.

(iv). Germ cell development: Primordial germ cells are specialized cells that give rise to gametes (sperm and eggs). A review paper presented in this Special Issue summarizes the latest findings on germ cell development, the methods available for obtaining germ cells in vitro, and their potential applications in canid species. 

(v). Reproductive disorders: New advances in disorders associated with reproduction were addressed in this Special Issue. Canine Brucellosis is a worldwide zoonosis, causing reproductive failures such as infertility and stillbirths [4]. The genomic characterization of virulence factors offers new information that is important for the development of diagnostic tools and effective vaccines for this infectious disease. Another disease commonly occurring in female dogs is mammary cancer, and one of the risk factors described for this pathology is the treatment with progestogens and estrogens [5]. A review paper presented in this Special Issue describes molecular mechanisms involved in the actions of estrogens that promote carcinogenic effects and disease progression. Canine mammary tumors are usually treated surgically. A study presented here has described the effect of pharmacological treatment using aglepristone prior to surgical intervention and reported that such treatment could reduce tumor size, but not density assessed by Shear Wave Elastography benign tumor models. Vaginitis is an inflammatory process of the vagina and is a common problem found in clinical practice [6]. A study examined the link between hematological parameters and vaginitis and reported the potential value of leukogram as a diagnostic tool for vaginitis. 

(vi). Pregnancy. Canine pregnancy is characterized by many specific features, three articles in this issue deal with different aspects of gestation and neonatal conditions. These include (i) a study describing fetal parameters associated with the length of gestation and their use as predictors of delivery time in the dog; (ii) maternal cardiovascular function assessment during pregnancy in Great Danes and the impact of the gestational stage on diastolic and systolic parameters; and (iii) a clinical evaluation of Apgar scores and umbilical cord blood gas analysis obtained from clamped umbilical cords of newborn pups delivered by the cesarean section.

(vii). Ovariohysterectomy. Excessive bleeding is the major complication during the ovariohysterectomy procedure. A study compared the ovarian artery flow velocity by a Doppler ultrasound before and after sedation with two alpha-2 agonists: medetomidine and dexmedetomidine. The authors found that the two anesthetic drugs decrease blood flow velocities in the ovarian artery, and thus, could be a useful option in ovariohysterectomy surgeries to reduce the risk of hemorrhages. 

Over the past few years, progress has been made toward a better understanding of mechanisms regulating canine reproduction, especially the aspect of gamete maturation and development. Further, studies have integrated molecular and imaging technologies to generate knowledge about disease progression and to develop diagnostic tools for reproductive disorders in domestic dogs.
